# Differences in radiographic and clinical outcomes of oblique lateral interbody fusion and lateral lumbar interbody fusion for degenerative lumbar disease: a meta-analysis

**DOI:** 10.1186/s12891-019-2972-7

**Published:** 2019-12-04

**Authors:** Hui-Min Li, Ren-Jie Zhang, Cai-Liang Shen

**Affiliations:** Department of Orthopedics & Spine Surgery, the First Affiliated Hospital of Anhui Medical University, 210 Jixi Road, Hefei, 230022 Anhui China

**Keywords:** Degenerative lumbar disease, OLIF, LLIF, XLIF, DLIF

## Abstract

**Background:**

In the current surgical therapeutic regimen for the degenerative lumbar disease, both oblique lateral interbody fusion (OLIF) and lateral lumbar interbody fusion (LLIF) are gradually accepted. Thus, the objective of this study is to compare the radiographic and clinical outcomes of OLIF and LLIF for the degenerative lumbar disease.

**Methods:**

We conducted an exhaustive literature search of MEDLINE, EMBASE, and the Cochrane Library to find the relevant studies about OLIF and LLIF for the degenerative lumbar disease. Random-effects model was performed to pool the outcomes about disc height (DH), fusion, operative blood loss, operative time, length of hospital stays, complications, visual analog scale (VAS), and Oswestry disability index (ODI).

**Results:**

56 studies were included in this study. The two groups of patients had similar changes in terms of DH, operative blood loss, operative time, hospital stay and the fusion rate (over 90%). The OLIF group showed slightly better VAS and ODI scores improvement. The incidence of perioperative complications of OLIF and LLIF was 26.7 and 27.8% respectively. Higher rates of nerve injury and psoas weakness (21.2%) were reported for LLIF, while higher rates of cage subsidence (5.1%), endplate damage (5.2%) and vascular injury (1.7%) were reported for OLIF.

**Conclusions:**

The two groups are similar in terms of radiographic outcomes, operative blood loss, operative time and the length of hospital stay. The OLIF group shows advantages in VAS and ODI scores improvement. Though the incidence of perioperative complications of OLIF and LLIF is similar, the incidence of main complications is significantly different.

## Background

Lumbar interbody fusion has been recognized as a powerful surgical tool for lumbar degenerative disease, including degenerative disc disease, spondylolisthesis, disc herniation, and deformity [1, 2]. Traditional techniques, such as anterior lumbar interbody fusion and posterior/transforaminal lumbar interbody fusion, have been successful with high patient satisfaction and fusion rates [1]. However, complications, such as excessive blood loss, iatrogenic muscle and soft tissue injury, muscular denervation, cannot be avoided [3]. To decrease surgical trauma, reduce operative bleeding and reduce hospital stay, minimally invasive spine (MIS) techniques such as oblique lateral interbody fusion (OLIF), lateral lumbar interbody fusion (LLIF), also known as extreme/direct lumbar interbody fusion (XLIF / DLIF), is progressively gaining popularity [3–7]. The advantages of both XLIF and OLIF are potential to restore both segmental and global lordosis, the latter often possible with targeting multiple levels through a single incision with minimally invasive surgery. Another advantage is the possibility of indirect decompression, avoiding the need in many cases to open spinal canal or foramina directly. Although radiographic and clinical outcomes of LLIF or OLIF have been assessed in many studies, however, few studies have compared the results of OLIF and LLIF. In the present study, considering the increasing interest in these techniques, our meta-analysis is performed to find differences in the radiographic and clinical outcomes of OLIF and LLIF for degenerative lumbar disease and, thus, provide vital evidence-based guidance for clinicians.

## Methods

### Literature review

**Fig. 1:**
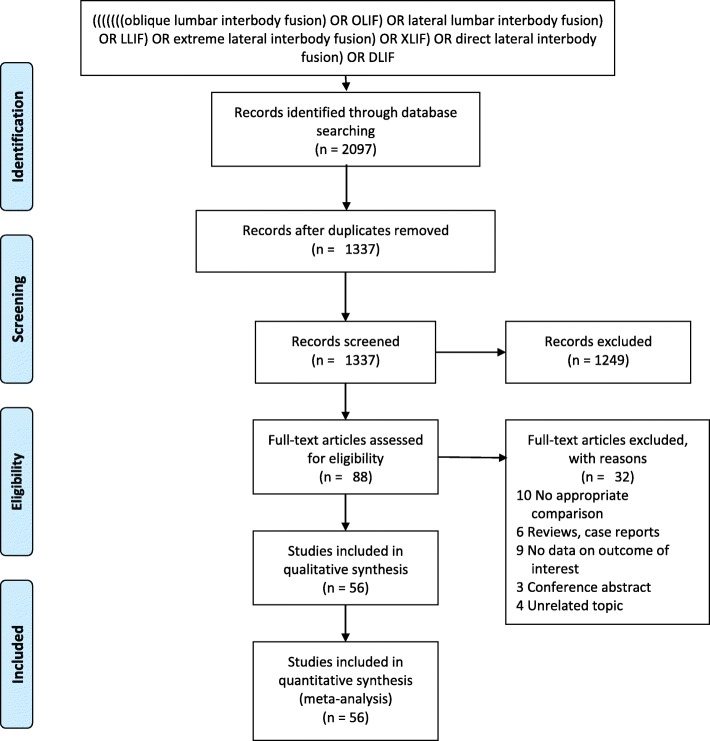
Flow diagram of study selection

MEDLINE (1966 to May 1, 2019), Embase (1974 to May 1, 2019) and Cochrane (2003 to May 1, 2019) were searched to find relevant studies about OLIF or LLIF for treatment for the degenerative lumbar disease. Studies were eligible for inclusion if they met the following criteria: studies reported outcomes at least one of the following outcomes: disc height (DH), visual analog scale (VAS), Oswestry disability index (ODI), operative blood loss, operative time, length of hospital stay, fusion, and complications; and had sufficient data to extract and pool. The following search terms were used: “oblique lumbar interbody fusion” or “OLIF” or “lateral lumbar interbody fusion” or “LLIF” or “extreme lateral interbody fusion” or “XLIF” or “direct lateral interbody fusion” or “DLIF”. We have found 2097 results through the above databases. Two of the reviewers (H.M. L. and R.J. Z.) independently examined the data extraction using standardized data extraction forms. There were altogether 1337 potentially relevant studies identified from the electronic search, the abstracts, and full manuscripts were also reviewed. The reference list of all relevant retrieved manuscripts was searched manually to identify additional studies that might have been missed. A summary of the study selection process in accordance with the Preferred Reporting Items for Systematic Reviews and Meta-Analyses guidelines was showed in Fig. 1.

### Study design

This study involved 56 studies, including 21 on OLIF [2, 3, 7–25] and 36 on LLIF [4–6, 10, 26–57] were relevant and analyzed in this study. The publishing year of these studies was between 2009 and 2019. Three studies were prospective consecutive clinical series, 16 studies were retrospective comparative studies, and 37 studies were retrospective case series. Two reviewers, using standardized data collection tool, independently extracted data on age, publication year, follow-up, fusion level, disc height (DH), visual analog scale (VAS), Oswestry disability index (ODI), operative blood loss, operative time, length of hospital stay, fusion, and complications [58]. Fusion was defined as bridging trabecular bone and lack of lucencies on plain radiographs and radiographic evidence of fusion was based on CT scans or flexion-extension radiographs. Any disagreements were resolved by discussion and consensus with a third party.

### Statistical methods

When pooling the data from the included studies, the Stata software (version 14.0; TX 77845, USA) was used. The z-test was applied to the mantel-haenszel analysis, and standardized mean difference (SMD) and 95% confidence interval (CI) was estimated by the random-effects model [58]. The heterogeneity was assessed using the chi-square test. The potential effects of mean age and follow-up on radiological and clinical outcomes were assessed by performing meta-regression. A *p*-value of 0.05 or less was considered statistically significant.

## Results

### Description of study

We have included fifty-six studies involving 2852 patients in this analysis. A total of 1304 patients aged 54.1–69 years (62.1 years on average) were included in the OLIF group. Follow-up was reported from 1 month to 24 months (10.5 months on average). Fusion levels have been reported to involve T10-T11 to L5-S1 (1 level: 154 patients; 2 levels: 192 patients, and ≥ 3 levels: 80 patients). A total of 1548 patients aged 51–74 years (62.2 years on average) were included in the LLIF group. Follow-up duration has been reported to range from 3 months to 35.4 months (17.7 months on average). Fusion levels have been reported to involve T12-L1 to L4-L5 (1 level: 278 patients, 2 levels: 139 patients, and ≥ 3 levels: 142 patients).

### Disc height

**Fig. 2:**
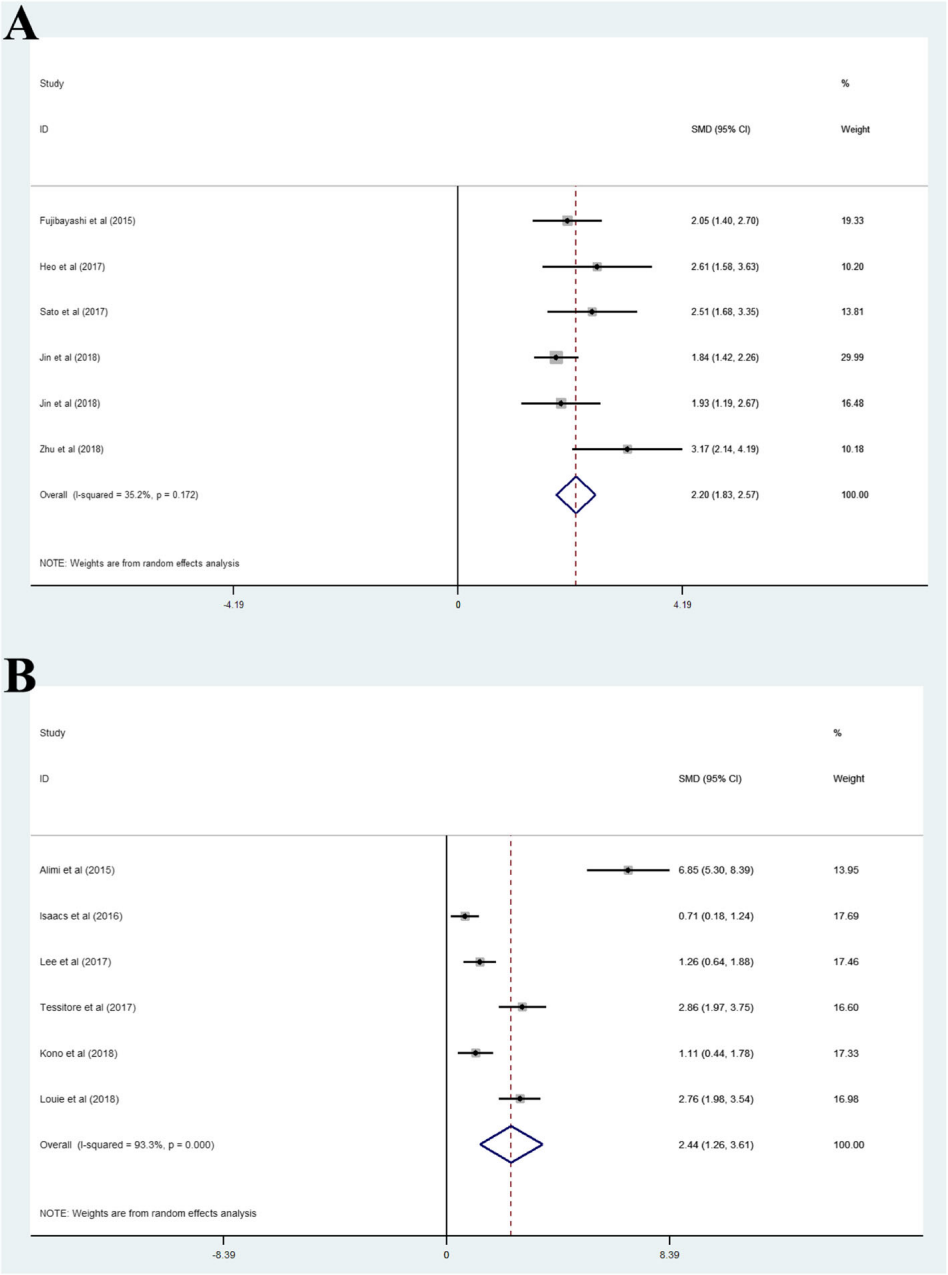
(A) Oblique lateral interbody fusion group, (B) lateral lumbar interbody fusion group. The positive and negative values of SMD represent disc height restoration and loss, respectively, after surgery. SMD, standard mean difference

Pooled analysis of six studies treated with OLIF [2, 8, 10, 14, 16, 23] showed that the pooled mean of DH did not vary across studies (I^2^ = 35.2%), and the pooled SMD was 2.20 (95% CI, 1.83–2.57). Pooled analysis of six studies treated with LLIF [5, 30, 31, 37, 43, 48] showed that the pooled mean of DH varies across studies (I^2^ = 93.3%), and the pooled SMD was 2.44 (95% CI, 1.26–3.61) (Fig. 2a and b).

### Visual analog scale

**Fig. 3:**
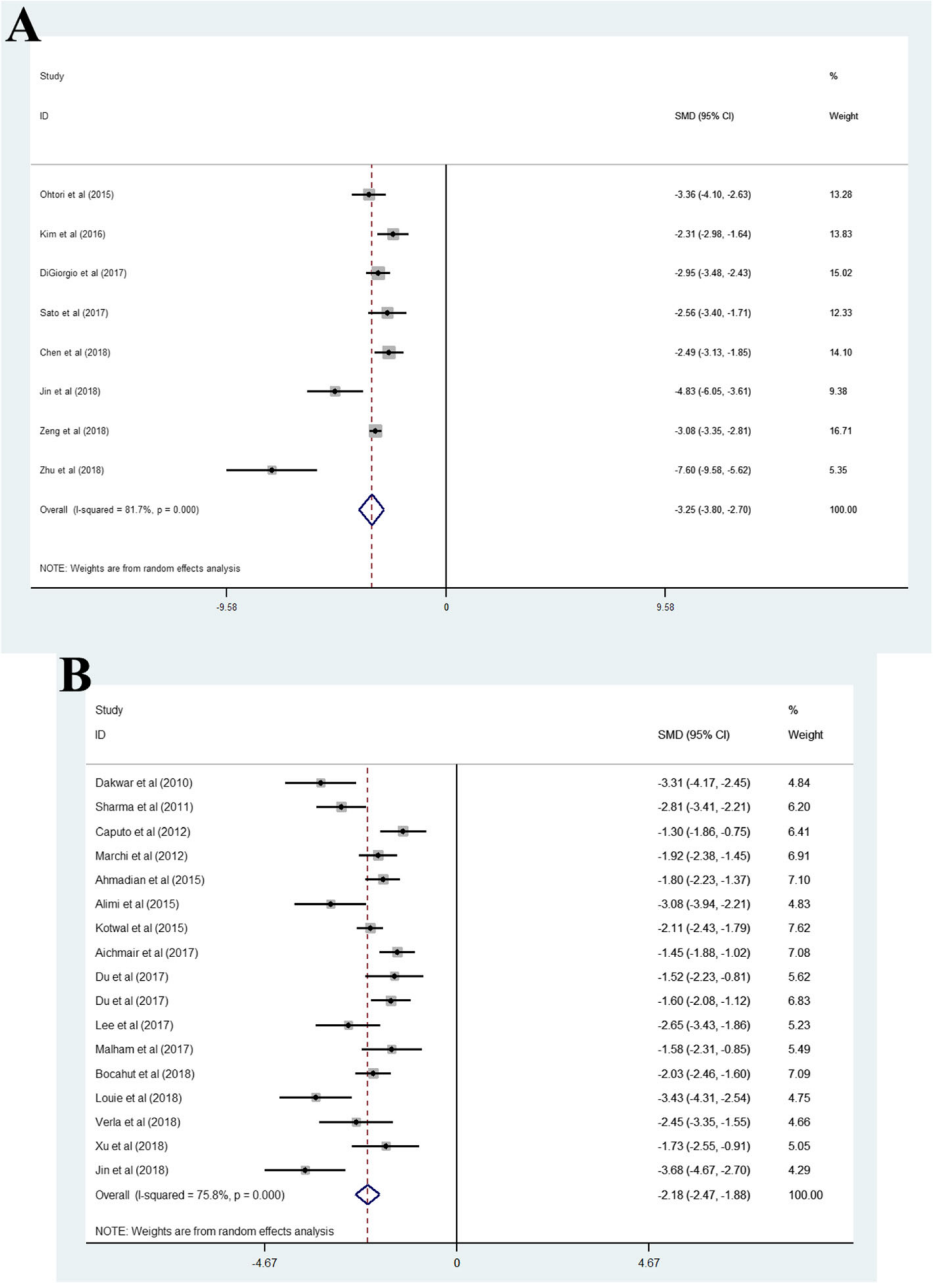
(A) Oblique lateral interbody fusion group, (B) lateral lumbar interbody fusion group. The negative and positive values of SMD represent visual analogue scale improvement and loss, respectively, after surgery. SMD, standard mean difference

Pooled analysis of eight studies treated with OLIF [3, 8–11, 14, 17, 22] showed that the pooled mean of VASs varies across studies (I^2^ = 91.7%), and the pooled SMD was − 3.25 (95% CI, − 3.80 to − 2.70). Pooled analysis of seventeen studies treated with LLIF [26, 27, 30, 33, 36–40, 46, 48, 49, 52] showed that the pooled mean of VASs varies across studies (I^2^ = 75.8%), and the pooled SMD was − 2.18 (95% CI, − 2.47 to − 1.88) (Fig. 3a and b).

### Oswestry disability index

**Fig. 4:**
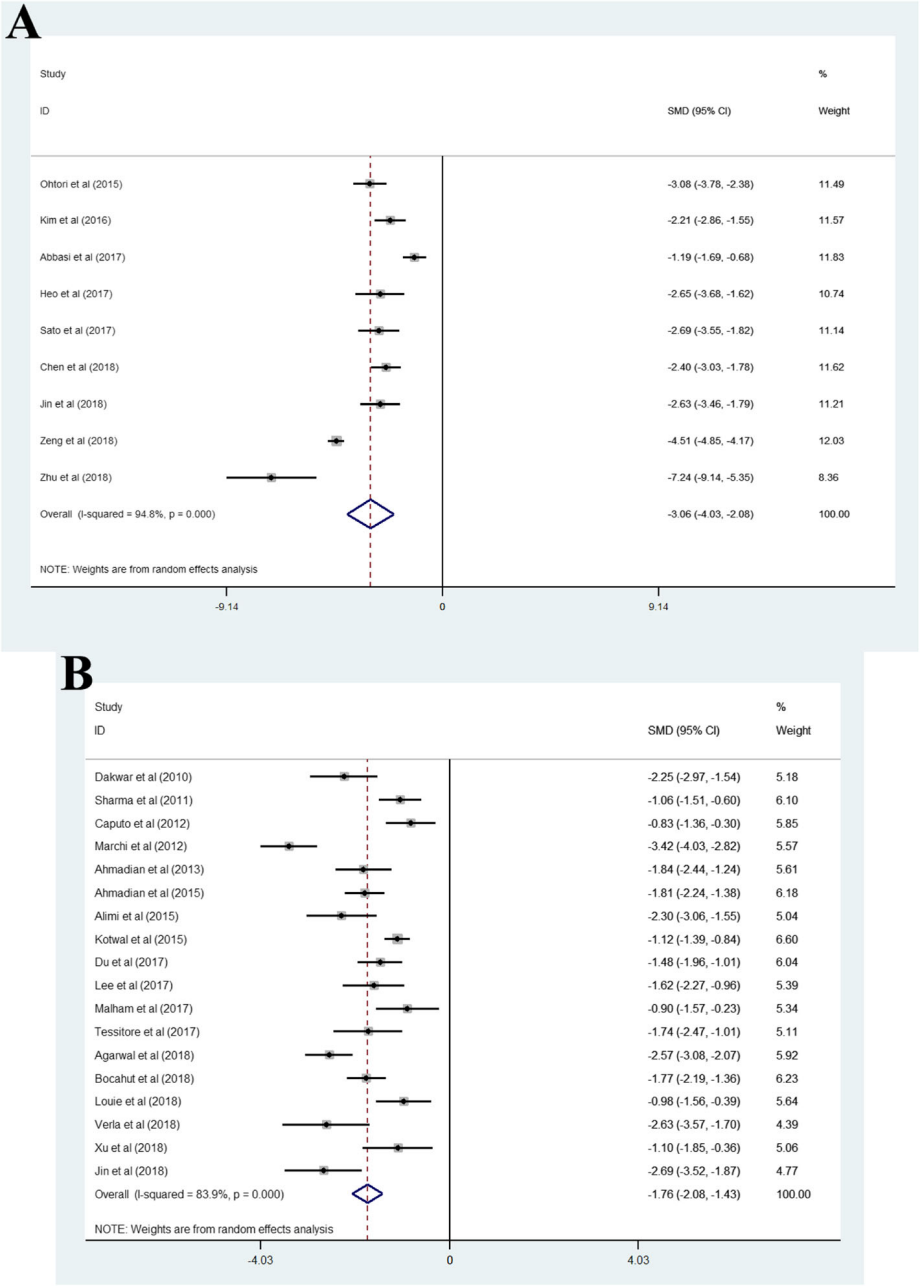
(A) Oblique lateral interbody fusion group, (B) lateral lumbar interbody fusion group. The negative and positive values of SMD represent Oswestry disability index improvement and loss, respectively, after surgery. SMD, standard mean difference

Pooled analysis of nine studies treated with OLIF [3, 8–11, 14, 16, 21, 22] showed that the pooled mean of ODIs varies across studies (I^2^ = 94.8%), and the pooled SMD was − 3.06 (95% CI, − 4.03 to − 2.08). Pooled analysis of eighteen studies treated with LLIF [5, 10, 26, 27, 30, 33, 34, 36–38, 46, 48, 49, 51–54, 57] showed that the pooled mean of ODIs varies across studies (I^2^ = 83.9%), and the pooled SMD was − 1.76 (95% CI, − 2.08 to − 1.43) (Fig. 4a and b).

### Operative blood loss, operative time, and length of hospital stay

Operative blood loss, operative time, and length of hospital stay have been reported in eleven studies of OLIF [2, 8–11, 16, 17, 21, 23–25] including 787 patients. OLIF combined with posterior fixation resulted in an average blood loss of 136 mL (49.7 for 1 operated level; 112.5 for 2 operated levels, 148.7 for 3 operated levels or more), an average operative time of 114 min (54.5 for 1 operated level; 67.2 for 2 operated levels, 119.5 for 3 operated levels or more), and an average length of hospital stay of 6.8 d (5.1 for 1 operated level; 6.4 for 2 operated levels, 7.9 for 3 operated levels or more) [58]. OLIF stand-alone [8, 11] resulted in an average operative blood loss of 83 mL, an average operative time of 104.2 min, and an average length of hospital stay of 6 d. Operative blood loss, operative time, and length of hospital stay have been reported in 29 studies of LLIF [6, 10, 26–32, 34, 35, 37–42, 44–46, 49, 51, 52, 54–57] including 1698 patients. LLIF combined with posterior fixation resulted in an average operative blood loss of 176 mL (71.6 for 1 operated level; 115 for 2 operated levels, 176 for 3 operated levels or more), an average operative time of 188 min (134.8 for 1 operated level; 174 for 2 operated levels, 253 for 3 operated levels or more), and an average length of hospital stay of 5.9 d (2 for 1 operated level; 3 for 2 operated levels, 4 for 3 operated levels or more). LLIF stand-alone [6, 30, 34, 40, 42, 46, 49, 52] resulted in an average operative blood loss of 76.4 mL, an average operative time of 126.2 min, and an average length of hospital stay of 5 d (Table 1).

**Table 1:** Operative blood loss, operative time and length of hospital stay of the study

	Operative blood loss (ml)				Operative time (min)				length of hospital stays (days)			
Fusion Level	OLIF + Posterior Fixation	OLIF Alone	LLIF + Posterior Fixation	LLIF Alone	OLIF + Posterior Fixation	OLIF Alone	LLIF + Posterior Fixation	LLIF Alone	OLIF + Posterior Fixation	OLIF Alone	LLIF + Posterior Fixation	LLIF Alone
1	49.7	NR	71.6	NR	54.5	NR	134.8	NR	5.1	NR	2	NR
2	112.5	NR	115	NR	67.2	NR	174	NR	6.4	NR	3	NR
≥3	148.7	NR	176	NR	119.5	NR	253	NR	7.9	NR	4	NR
Mean	136	83	176	76.4	114	104.2	188	126.2	6.8	6	5.9	5

*OLIF* oblique lateral interbody fusion; *LLIF* lateral lumbar interbody fusion; *NR* not reported

### Fusion results and complications

Five studies about OLIF [8, 12, 19, 21, 22] with a total of 287 patients and fusion was reported in 278 patients. Twelve studies about LLIF [30, 38–40, 46, 47, 49, 51, 52, 54, 55, 57] with a total of 584 patients and fusion was reported in 535 patients.

Seventeen studies about OLIF [2, 3, 7–10, 12, 14–23, 25] with a total of 1043 patients reported an incidence of perioperative complications of 26.7%. The most common perioperative complication was thigh pain/numbness or psoas weakness (8.8%). Other main perioperative complications included endplate fracture (5.2%), cage subsidence (5.1%), vascular injury (1.7%) and the details of perioperative complications of OLIF are shown in Table 3. Thirty-two studies about LLIF [4–6, 10, 26–32, 34, 35, 37–42, 44–47, 49–57] with a total of 1562 patients reported an incidence of perioperative complications of 27.8%. The most common perioperative complication was thigh pain/numbness or psoas weakness (21.2%). Other main perioperative complications included cage subsidence (1.3%) and the details of perioperative complications of LLIF are shown in Table 2.

**Table 2:** Comparison of clinical complications between both groups

	OLIF Alone [8] (*n* = 17)	OLIF + Posterior Fixation (*n* = 1026)	OLIF Total (*n* = 1043)	LLIF Alone [6, 30, 34, 49, 52] (*n* = 249)	LLIF + Posterior Fixation (*n* = 1313)	LLIF Total (*n* = 1562)
vascular complications	0	18	18	0	0	0
Neurological injury	0	3	3	2	1	3
thigh pain/numbness, psoas weakness						
permanent	0	1	1	0	4	4
impermanent	1	90	91	50	277	327
gastrointestinal and urinary complications	0	26	26	1	21	22
wound complications						
infections	0	3	3	0	11	11
Hematoma	0	4	4	0	4	4
Seroma	0	1	1	0	1	1
wound pain	2	3	5	0	1	1
Revision Surgery	0	12	12	3	10	13
Hardware failure	0	4	4	0	1	1
Failure of Operation						
EndPlate Fracture	0	54	54	0	2	2
Vertebral fracture	0	2	2	0	3	3
Cage subsidence	0	53	53	15	5	20
Rupture of ALL	0	0	0	0	2	2
complications not directly related to surgical access						
pulmonary/ respiratory	0	1	1	2	1	3
cardiac	0	1	1	7	10	17

*OLIF* oblique lateral interbody fusion; *LLIF* lateral lumbar interbody fusion; *ALL* anterior longitudinal ligament

### Meta-regression analyses and publication bias

Results of meta-regression demonstrated that the follow-up period had no effect on DH, VAS, or ODI, and mean age had no effect on DH or VAS in both surgical groups, However, the mean age was negatively correlated with decreasing ODI score in the OLIF group (Table 3).

**Table 3:** Meta-regression of potential effect of follow-up and mean age on the radiographic and clinical indexes

	Follow-up				Mean age			
	OLIF		LLIF		OLIF		LLIF	
	Coefficient (95% CI)	*P > t*	Coefficient (95% CI)	*P > t*	Coefficient (95% CI)	*P > t*	Coefficient (95% CI)	*P > t*
Disc height	0.01(−0.79 to 0.09)	0.88	−0.07(−0.31 to 0.16)	0.44	−0.09(−0.32 to 0.15)	0.36	0.11(−0.67 to 0.89)	0.73
Visual analogue scale	0.01(− 0.23 to 0.24)	0.94	−0.01(− 0.05 to 0.04)	0.84	0.36(− 0.05 to 0.78)	0.08	− 0.04(− 0.17 to 0.09)	0.54
Oswestry Disability Index	−0.08(− 0.28 to 0.12)	0.38	0.02(− 0.02 to 0.06)	0.34	0.46 (0.18 to 0.74)	0.01*	−0.06(− 0.15 to 0.02)	0.14

*OLIF* oblique lateral interbody fusion; *LLIF* lateral lumbar interbody fusion; ^*^P < 0.05 is considered as the factor that contributes to the heterogeneity of effect. If coefficient interval goes across both negative and positive values, it means that no evidence supports that the factor contributes significantly to the heterogeneity of effect

**Fig. 5:**
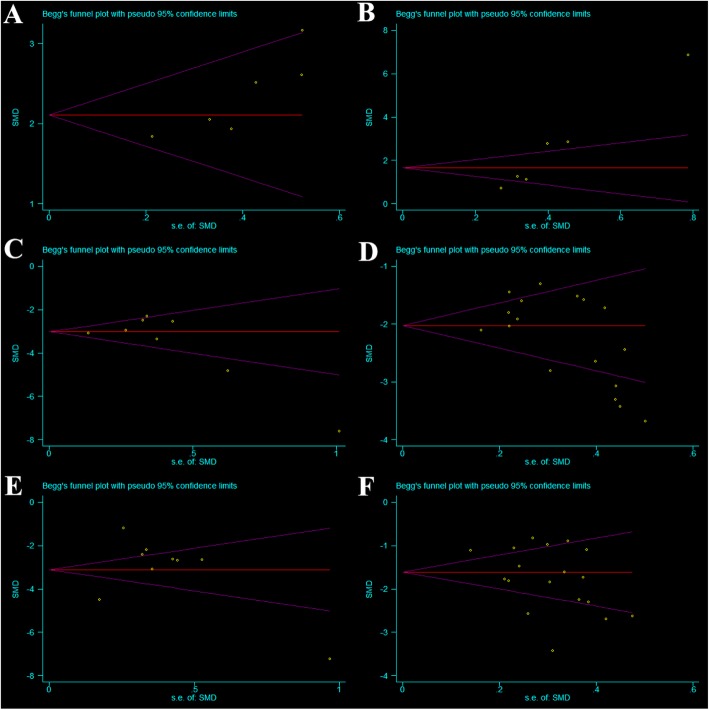
Funnel plot of publication bias. (**a**) Disc height of oblique lateral interbody fusion (OLIF), (**b**) disc height of lateral lumbar interbody fusion (LLIF), (**c**) visual analog scale of OLIF, (**d**) visual analog scale of LLIF, (**e**) Oswestry disability index of OLIF, and (**f**) Oswestry disability index of LLIF. No publication bias for DH or ODI was found in either group. Publication bias was found for VAS for the LLIF group

No publication bias for DH or ODI was observed in either group, but publication bias for VAS was observed for the LLIF group (Fig. 5).

## Discussion

Our meta-analysis was designed to explore differences in radiographic and clinical outcomes of OLIF and LLIF. The importance of restoring sagittal balance and lumbar lordosis is increasingly recognized in any spinal reconstruction surgery. The most important factor affecting lumbar lordosis recovery is DH restoration [59]. In the present study, very similar results about restorations of DH (2.20 versus 2.44) were observed between the OLIF and LLIF techniques. Most studies have shown OLIF and LLIF could restore the DH [8, 43, 48], and some researchers have found that the OLIF group showed a significant increase of posterior disk space compared with the LLIF group (40.2% vs. 31.6%, respectively), which may be explained by the fact that the orthogonal maneuver provides the controllability of cage position, and earlier change of direction from oblique to direct lateral makes the anterior position of the cage to obtain a large segmental angle for the kyphosis correction [10]. However, other study demonstrated that OLIF might make no difference in restoring the DH, as more than half of the patients without applying posterior supplemental fixation in their study [11]. And considering the heterogeneity of this meta-analysis, meta-regression was applied for exploring the cause of heterogeneity of the potential effect of mean age and follow-up on DH restoration, but we found that the follow-up period and mean age did not affect DH in both groups.

According to previous studies, both OLIF and LLIF presented lower postoperative VAS and ODI scores compared with preoperative values [8, 9, 38, 39], which is consistent with our results. Furthermore, we have found the postoperative VAS and ODI scores in the OLIF group were better than those in the LLIF group (− 3.25 versus − 2.18; − 3.06 versus − 1.76, respectively), probably due to complications related to the psoas muscle injury in the LLIF group. Besides, meta-regression has shown that the mean age was negatively correlated with decreasing ODI score in the OLIF group.

This meta-analysis suggests that OLIF showed similar operative blood loss, operative time, and hospital stay than those compared with LLIF. This may be because the OLIF and LLIF techniques are performed through the retroperitoneal space and by blunt dissection. Besides, the number of manipulations during surgery is decreased especially when radiographs are required, as no microscope or other specific complex ancillary is used in both groups.

The meta-analysis also suggests that OLIF achieves a similar fusion rate (> 90%) compared with that of LLIF. However, a high rate of perioperative complications was observed related to OLIF and LLIF, and there were differences between the two groups. Higher rates of nerve injury and psoas weakness were reported for LLIF, while higher rates of cage subsidence, endplate damage, and vascular injury were reported for OLIF. Direct injury to the lumbar plexus is the greatest risk from LLIF, as it has the potential to result in sensory and motor deficits. According to previous studies, the incidence of thigh dysfunction and numbness after LLIF has been reported to range from 0.7 to 30%, and the incidence of motor weakness ranged from 3.4 to 23.7% [6, 47, 60, 61]. In our study, we have found the incidence of anterior thigh numbness and leg weakness was high at 21.2%. Although most of those symptoms are transient and completely recovered by time in most studies, there are still some studies reported that some patients presented with the permanent damages [10, 62]. The possible reason for this phenomenon may be ascribed to direct injury to lumbar plexus, ilioinguinal nerve or hypogastric nerve that are situated within the abdominal wall, and injury to the genitofemoral nerve on the psoas muscle during the surgical approach. Although thigh pain/numbness and psoas weakness were observed in OLIF group, the incidence of these complications was lower compared to LLIF. However, higher rates of cage subsidence (5.1%), endplate damage (5.2%) and vascular injury (1.7%) were reported for OLIF. The presence of a damaged endplate, an embedded cage, cage sedimentation, and shifting correlated highly, mainly because of endplate lesions [58]. The high risk of cage subsidence after OLIF may be also due to the smaller interbody grafts limited by the discectomy corridor [61]. Besides, vascular injury was a noteworthy complication of OLIF, and the incidence of vascular injury has been reported to range from 1.1 to 2.8% [3, 25, 61]. Although the incidence of major vascular injury was low, careful preoperative radiological evaluation of vascular anatomy should be conducted to avoid these complications [62].

One limitation of this review is that most of the studies included were retrospective studies with incomplete outcome reporting. Secondly, we did not compare segmental lordosis, global lordosis as more studies need to be included. Thirdly, the fusion result of both techniques may be influenced by the kind of graft material. Another limitation is that all the included studies are searched through an online database but not included unpublished studies, which might have led to publication bias in our meta-analysis. Finally, the interpretation of thigh symptoms was controversial, as surgeons have gradually realized that thigh symptoms disappeared spontaneously 2–3 weeks after surgery, so the definition of this symptom was changed to recognize it as an access-related symptom but not as a complication. However, any meta-analysis has the risk of publication, and we believe that our final result is convincing.

## Conclusions

The two groups are similar in terms of radiographic outcomes, operative blood loss, operative time and the length of hospital stay. The OLIF group shows advantages in VAS and ODI scores improvement. Though the incidence of perioperative complications of OLIF and LLIF is similar, the incidence of main complications is significantly different.

## Data Availability

The datasets used and/or analysed during the current study are available from the corresponding author on reasonable request.

## Abbreviations

DH: Disc height
DLIF: Direct lumbar interbody fusion
LLIF: Lateral lumbar interbody fusion
ODI: Oswestry Disability Index
OLIF: Oblique lateral interbody fusion
VAS: Visual analog scale
XLIF: Extreme lumbar interbody fusion

## References

[1] Wang HW, Hu YC, Wu ZY, Wu HR, Wu CF, Zhang LS, Xu WK, Fan HL, Cai JS, Ma JQ (2017). Minimally invasive Transforaminal lumbar Interbody fusion and unilateral fixation for degenerative lumbar disease. Orthop Surg.

[2] Jin C, Jaiswal MS, Jeun SS, Ryu KS, Hur JW, Kim JS (2018). Outcomes of oblique lateral interbody fusion for degenerative lumbar disease in patients under or over 65 years of age. J Orthop Surg Res.

[3] Ohtori S, Orita S, Yamauchi K, Eguchi Y, Ochiai N, Kishida S, Kuniyoshi K, Aoki Y, Nakamura J, Ishikawa T (2015). Mini-open anterior retroperitoneal lumbar Interbody fusion: oblique lateral Interbody fusion for lumbar spinal degeneration disease. Yonsei Med J.

[4] Sellin JN, Brusko GD, Levi AD (2019). Lateral lumbar Interbody fusion revisited: complication avoidance and outcomes with the mini-open approach. World Neurosurg.

[5] Tessitore E, Molliqaj G, Schaller K, Gautschi OP (2017). Extreme lateral interbody fusion (XLIF): a single-center clinical and radiological follow-up study of 20 patients. J clinical neurosci : official j Neurosurg Society Australas.

[6] Knight RQ, Schwaegler P, Hanscom D, Roh J (2009). Direct lateral lumbar interbody fusion for degenerative conditions: early complication profile. J Spinal Disord Tech.

[7] Miscusi M, Ramieri A, Forcato S, Giuffre M, Trungu S, Cimatti M, Pesce A, Familiari P, Piazza A, Carnevali C (2018). Comparison of pure lateral and oblique lateral inter-body fusion for treatment of lumbar degenerative disk disease: a multicentric cohort study. Eur spine j : official publication Eur Spine Soc, Eur Spinal Deformity Soc, Eur Section Cervical Spine Res Soc.

[8] Zhu G, Hao Y, Yu L, Cai Y, Yang X (2018). Comparing stand-alone oblique lumbar interbody fusion with posterior lumbar interbody fusion for revision of rostral adjacent segment disease: a STROBE-compliant study. Medicine.

[9] Zeng ZY, Xu ZW, He DW, Zhao X, Ma WH, Ni WF, Song YX, Zhang JQ, Yu W, Fang XQ (2018). Complications and prevention strategies of oblique lateral Interbody fusion technique. Orthop Surg.

[10] Jin J, Ryu KS, Hur JW, Seong JH, Kim JS, Cho HJ (2018). Comparative study of the difference of perioperative complication and radiologic results: MIS-DLIF (minimally invasive direct lateral lumbar Interbody fusion) versus MIS-OLIF (minimally invasive oblique lateral lumbar Interbody fusion). Clinical spine surg.

[11] Chen YL, Zhu ZH, Wang YK, Fan SW, Fang XQ, Wan SL, Zhang JF, Zhao X, Zhao FD: [effects of oblique lateral interbody fusion and transforaminal lumbar interbody fusion for lordosis correction in degenerative lumbar diseases]. Zhonghua Yi Xue Za Zhi 2018, 98(25):1990–1995. 10.3760/cma.j.issn.0376-2491.2018.25.005 29996598

[12] Woods KR, Billys JB, Hynes RA (2017). Technical description of oblique lateral interbody fusion at L1-L5 (OLIF25) and at L5-S1 (OLIF51) and evaluation of complication and fusion rates. Spine j official j North American Spine Soc.

[13] Woods K, Fonseca A, Miller LE (2017). Two-year outcomes from a single Surgeon's learning curve experience of oblique lateral Interbody fusion without intraoperative Neuromonitoring. Cureus.

[14] Sato J, Ohtori S, Orita S, Yamauchi K, Eguchi Y, Ochiai N, Kuniyoshi K, Aoki Y, Nakamura J, Miyagi M (2017). Radiographic evaluation of indirect decompression of mini-open anterior retroperitoneal lumbar interbody fusion: oblique lateral interbody fusion for degenerated lumbar spondylolisthesis. Eur spine j official publication Eur Spine Soc Eur Spinal Deformity Soc Eur Section Cervical Spine Res Soc.

[15] Hur JW, Ryu KS, Kim JS, Seong JH, Cho HJ, Chung HJ (2017). Oral Presentations. Global spine j.

[16] Heo DH, Kim JS (2017). Clinical and radiological outcomes of spinal endoscopic discectomy-assisted oblique lumbar interbody fusion: preliminary results. Neurosurg Focus.

[17] DiGiorgio AM, Edwards CS, Virk MS, Mummaneni PV, Chou D (2017). Stereotactic navigation for the prepsoas oblique lateral lumbar interbody fusion: technical note and case series. Neurosurg Focus.

[18] Chung NS, Jeon CH, Lee HD, Seo YU (2017). EUROSPINE 2017 Scientific Programme Quick Fire presentations. Eur spine j official publication Eur Spine Soc Eur Spinal Deformity Soc Eur Section Cervical Spine Res Soc.

[19] Choi WS, Cho HJ, Lim KH, Kim JS (2017). E-Posters. Global spine j.

[20] Abe K, Orita S, Mannoji C, Motegi H, Aramomi M, Ishikawa T, Kotani T, Akazawa T, Morinaga T, Fujiyoshi T (2017). Perioperative complications in 155 patients who underwent oblique lateral interbody fusion surgery perspectives and indications from a retrospective, multicenter survey. Spine vol.

[21] Abbasi H, Miller L, Abbasi A, Orandi V, Khaghany K (2017). Minimally invasive scoliosis surgery with oblique lateral lumbar Interbody fusion: single surgeon feasibility study. Cureus.

[22] Kim J-S, Choi WS, Sung JH (2016). 314 minimally invasive oblique lateral Interbody fusion for L4-5. Neurosurg.

[23] Fujibayashi S, Hynes RA, Otsuki B, Kimura H, Takemoto M, Matsuda S (2015). Effect of indirect neural decompression through oblique lateral interbody fusion for degenerative lumbar disease. Spine.

[24] Abbasi H, Abbasi A (2015). Oblique lateral lumbar Interbody fusion (OLLIF): technical notes and early results of a single surgeon comparative study. Cureus.

[25] Silvestre C, Mac-Thiong JM, Hilmi R, Roussouly P (2012). Complications and morbidities of mini-open anterior retroperitoneal lumbar Interbody fusion: oblique lumbar Interbody fusion in 179 patients. Asian spine j.

[26] Xu DS, Bach K, Uribe JS (2018). Minimally invasive anterior and lateral transpsoas approaches for closed reduction of grade II spondylolisthesis: initial clinical and radiographic experience. Neurosurg Focus.

[27] Verla T, Winnegan L, Mayer R, Cherian J, Yaghi N, Palejwala A, Omeis I (2018). Minimally invasive Transforaminal versus direct lateral lumbar Interbody fusion: effect on return to work, narcotic use, and quality of life. World Neurosurg.

[28] Tamburrelli FC, Meluzio MC, Burrofato A, Perna A, Proietti L (2018). Minimally invasive surgery procedure in isthmic spondylolisthesis. Eur spine j official publication Eur Spine Soc Eur Spinal Deformity Soc Eur Sect Cervical Spine Res Soc.

[29] Panchal R, Denhaese R, Hill C, Strenge KB, DEM A, Passias P, Arnold P, Cappuccino A, Dennis MD, Kranenburg A (2018). Anterior and lateral lumbar Interbody fusion with supplemental Interspinous process fixation: outcomes from a multicenter, prospective, randomized, controlled study. Int j spine surg.

[30] Louie PK, Varthi AG, Narain AS, Lei V, Bohl DD, Shifflett GD, Phillips FM (2018). Stand-alone lateral lumbar interbody fusion for the treatment of symptomatic adjacent segment degeneration following previous lumbar fusion. Spine j official j North American Spine Soc.

[31] Kono Y, Gen H, Sakuma Y, Koshika Y (2018). Comparison of clinical and radiologic results of mini-open Transforaminal lumbar Interbody fusion and extreme lateral Interbody fusion indirect decompression for degenerative lumbar Spondylolisthesis. Asian spine j.

[32] Campbell PG, Nunley PD, Cavanaugh D, Kerr E, Utter PA, Frank K, Stone M (2018). Short-term outcomes of lateral lumbar interbody fusion without decompression for the treatment of symptomatic degenerative spondylolisthesis at L4-5. Neurosurg Focus.

[33] Bocahut N, Audureau E, Poignard A, Delambre J, Queinnec S, Flouzat Lachaniette CH, Allain J (2018). Incidence and impact of implant subsidence after stand-alone lateral lumbar interbody fusion. Orthop and traumatol surg and res OTSR.

[34] Agarwal N, Faramand A, Alan N, Tempel ZJ, Hamilton DK, Okonkwo DO, Kanter AS (2018). Lateral lumbar interbody fusion in the elderly: a 10-year experience. J neurosurg Spine.

[35] Pereira EA, Farwana M, Lam KS (2017). Extreme lateral interbody fusion relieves symptoms of spinal stenosis and low-grade spondylolisthesis by indirect decompression in complex patients. J clinical neurosci official j Neurosurg Soc Australasia.

[36] Malham GM, Ellis NJ, Parker RM, Blecher CM, White R, Goss B, Seex KA (2017). Maintenance of segmental Lordosis and disk height in stand-alone and instrumented extreme lateral Interbody fusion (XLIF). Clinical spine surg.

[37] Lee CW, Yoon KJ, Ha SS (2017). Which approach is advantageous to preventing development of adjacent segment disease? Comparative analysis of 3 different lumbar Interbody fusion techniques (ALIF, LLIF, and PLIF) in L4-5 Spondylolisthesis. World Neurosurg.

[38] Du JY, Kiely PD, Bogner E, Al Maaieh M, Aichmair A, Salzmann SN, Huang RC (2017). Early clinical and radiological results of unilateral posterior pedicle instrumentation through a Wiltse approach with lateral lumbar interbody fusion. J spine surg (Hong Kong).

[39] Du JY, Kiely PD, Al Maaieh M, Aichmair A, Huang RC (2017). Lateral lumbar interbody fusion with unilateral pedicle screw fixation for the treatment of adjacent segment disease: a preliminary report. J spine surg (Hong Kong).

[40] Aichmair A, Alimi M, Hughes AP, Sama AA, Du JY, Hartl R, Burket JC, Lampe LP, Cammisa FP, Girardi FP (2017). Single-level lateral lumbar Interbody fusion for the treatment of adjacent segment disease: a retrospective two-center study. Spine.

[41] Sembrano JN, Tohmeh A, Isaacs R, Group SDS (2016). Two-year comparative outcomes of MIS lateral and MIS Transforaminal Interbody fusion in the treatment of degenerative Spondylolisthesis: part I: clinical findings. Spine.

[42] Nunley P, Sandhu F, Frank K, Stone M (2016). Neurological complications after lateral Transpsoas approach to anterior Interbody fusion with a novel flat-blade spine-fixed retractor. Biomed Res Int.

[43] Isaacs RE, Sembrano JN, Tohmeh AG, Group SDS (2016). Two-year comparative outcomes of MIS lateral and MIS Transforaminal Interbody fusion in the treatment of degenerative Spondylolisthesis: part II: radiographic findings. Spine.

[44] Grimm BD, Leas DP, Poletti SC, Johnson DR (2016). Postoperative complications within the first year after extreme lateral Interbody fusion: experience of the first 108 patients. Clinical spine surg.

[45] Pawar AY, Hughes AP, Sama AA, Girardi FP, Lebl DR, Cammisa FP (2015). A comparative study of lateral lumbar Interbody fusion and posterior lumbar Interbody fusion in degenerative lumbar Spondylolisthesis. Asian spine j.

[46] Kotwal S, Kawaguchi S, Lebl D, Hughes A, Huang R, Sama A, Cammisa F, Girardi F (2015). Minimally invasive lateral lumbar Interbody fusion: clinical and radiographic outcome at a minimum 2-year follow-up. J Spinal Disord Tech.

[47] Berjano P, Langella F, Damilano M, Pejrona M, Buric J, Ismael M, Villafane JH, Lamartina C (2015). Fusion rate following extreme lateral lumbar interbody fusion. Eur spine j official publication Eur Spine Soci Eur Spinal Deformity Soc Eur Sec Cervical Spine Res Soc.

[48] Alimi M, Hofstetter CP, Tsiouris AJ, Elowitz E, Hartl R (2015). Extreme lateral interbody fusion for unilateral symptomatic vertical foraminal stenosis. Eur spine j official publication Eur Spine Soc Eur Spinal Deformity Soc Eur Sec Cervical Spine Res Soc.

[49] Ahmadian A, Bach K, Bolinger B, Malham GM, Okonkwo DO, Kanter AS, Uribe JS (2015). Stand-alone minimally invasive lateral lumbar interbody fusion: multicenter clinical outcomes. J clinical neurosci official j Neurosurg Soc Australasia.

[50] Hrabalek L, Adamus M, Gryga A, Wanek T, Tucek P (2014). A comparison of complication rate between anterior and lateral approaches to the lumbar spine. Biomed papers Med Faculty University Palacky, Olomouc, Czechoslovakia.

[51] Ahmadian A, Verma S, Mundis GM, Oskouian RJ, Smith DA, Uribe JS (2013). Minimally invasive lateral retroperitoneal transpsoas interbody fusion for L4-5 spondylolisthesis: clinical outcomes. J neurosurg Spine.

[52] Marchi L, Abdala N, Oliveira L, Amaral R, Coutinho E, Pimenta L (2012). Stand-alone lateral interbody fusion for the treatment of low-grade degenerative spondylolisthesis. Sci World J.

[53] Caputo AM, Michael KW, Chapman TM, Massey GM, Howes CR, Isaacs RE, Brown CR (2012). Clinical outcomes of extreme lateral interbody fusion in the treatment of adult degenerative scoliosis. Sci World J.

[54] Sharma AK, Kepler CK, Girardi FP, Cammisa FP, Huang RC, Sama AA (2011). Lateral lumbar interbody fusion: clinical and radiographic outcomes at 1 year: a preliminary report. J Spinal Disord Tech.

[55] Ozgur BM, Agarwal V, Nail E, Pimenta L (2010). Two-year clinical and radiographic success of minimally invasive lateral transpsoas approach for the treatment of degenerative lumbar conditions. SAS J.

[56] Isaacs RE, Hyde J, Goodrich JA, Rodgers WB, Phillips FM (2010). A prospective, nonrandomized, multicenter evaluation of extreme lateral interbody fusion for the treatment of adult degenerative scoliosis: perioperative outcomes and complications. Spine.

[57] Dakwar E, Cardona RF, Smith DA, Uribe JS (2010). Early outcomes and safety of the minimally invasive, lateral retroperitoneal transpsoas approach for adult degenerative scoliosis. Neurosurg Focus.

[58] Li HM, Zhang RJ, Shen CL (2019). Radiographic and clinical outcomes of oblique lateral Interbody fusion versus minimally invasive Transforaminal lumbar Interbody fusion for degenerative lumbar disease. World Neurosurg.

[59] Sembrano JN, Yson SC, Horazdovsky RD, Santos ER, Polly DW (2015). Radiographic comparison of lateral lumbar Interbody fusion versus traditional fusion approaches: analysis of sagittal contour change. Int j spine surg.

[60] Rodgers WB, Gerber EJ, Patterson J (2011). Intraoperative and early postoperative complications in extreme lateral interbody fusion: an analysis of 600 cases. Spine.

[61] Xu DS, Walker CT, Godzik J, Turner JD, Smith W, Uribe JS (2018). Minimally invasive anterior, lateral, and oblique lumbar interbody fusion: a literature review. Ann transl med.

[62] Fujibayashi S, Kawakami N, Asazuma T, Ito M, Mizutani J, Nagashima H, Nakamura M, Sairyo K, Takemasa R, Iwasaki M (2017). Complications associated with lateral Interbody fusion: Nationwide survey of 2998 cases during the first 2 years of its use in Japan. Spine.

